# Nutritional Knowledge and Dietary Intake Habits among Pregnant Adolescents Attending Antenatal Care Clinics in Urban Community in Ghana

**DOI:** 10.1155/2021/8835704

**Published:** 2021-02-13

**Authors:** Prince Kubi Appiah, Anang Rhoda Naa Korklu, Duut Abdulai Bonchel, Georgina Agartha Fenu, Francis Wadga-Mieza Yankey

**Affiliations:** ^1^Department of Family and Community Health, School of Public Health, University of Health and Allied Sciences, Ho, Ghana; ^2^Department of Medical Law and Ethics, Asian Institute for Bioethics and Health Law, College of Medicine, Yonsei University, Seoul, Republic of Korea; ^3^Centre for Migration Studies, University of Ghana, Legon, Accra, Ghana; ^4^Department of Epidemiology and Biostatistics, School of Public Health, University of Health and Allied Sciences, Ho, Ghana; ^5^Academic Affairs Directorate, University of Health and Allied Sciences, Ho, Ghana

## Abstract

**Introduction:**

Proper nutrition during pregnancy is important for the wellbeing of the mother and foetus and supports health during pregnancy, delivery, and breastfeeding. However, there are little data on nutritional knowledge and dietary intake among adolescents who are pregnant in Ghana. Hence, the study assessed the nutritional knowledge and eating habits of this vulnerable group in the Ledzokuku-Krowor Municipality, Ghana.

**Methods:**

The study was cross-sectional and employed a multistage sampling technique to select 423 participants. The study was conducted between October and November 2019. A statistical software was used to analyse data and employed Pearson's chi-square and logistics regression to assess associations between the outcome and predictor variables. A *p* value <0.05 at a 95% confidence interval was considered statistically significant.

**Results:**

Less than half (44.9%) of the pregnant adolescents have high nutritional knowledge. About 19.4% of them have good eating habits, while 23.9%, 18.2%, and 6.4% of them do not take breakfast, lunch, and supper, respectively. However, 15.6%, 13.9%, and 9.2% do take snacks after breakfast, lunch, and supper, respectively. About 55.9%, 59.8%, and 23.0% do not take their breakfast, lunch, and supper on time, respectively. Additionally, only 3.8% of them do take fruits and vegetables daily, while 9.7%, 23.2%, 30.0%, and 26.5% of them do take animal products, energy drinks, carbonated drinks, and legumes/nuts/seeds daily, respectively. The study showed that educational level (*p*=0.014), occupation (*p*=0.016), ethnicity (*p*=0.017), and number of pregnancies (*p*=0.021) were associated with good eating habits.

**Conclusion:**

Eating habit of adolescent pregnant women was not encouraging. Therefore, the municipal health authority with the concerned stakeholders should intensify efforts, including nutritional education to improve good eating habits, such as taking snacks in between meals, eating on time, and balance diet among pregnant adolescents, and to reduce adolescent pregnancy in the municipality.

## 1. Introduction

Adolescent pregnancy is a key public health obstacle in the world and has been linked with substantial medical, nutritional, social, and economic risks for individuals, the child, family, and communities [1]. Proper nutrition during pregnancy is necessary for maternal health during pregnancy, delivery, and breastfeeding [2] and influences the growth and development of the foetus [3]. Hence, an adequate amount of nutrients is needed to support foetal growth and development, along with the alterations in maternal tissues and metabolism [4]. Though nutrients requirement increases during pregnancy [5], it is known that intake of essential nutrients among adolescents is below what is recommended [6], with notable deficiencies of iron leading to cognitive and behavioural problems in childhood, zinc likely to limit foetal growth, vitamin A leading to impair resistance to infection, and calcium associated with preeclampsia and intrauterine growth restriction [7]. Meanwhile, adolescence is a life stage where there is rapid growth and development with a significant increase in nutritional requirements, and the additional nutrient demands of pregnancy subject these adolescents to greater health risks [8]. Again, poor pregnancy outcomes, including low birth weight and anaemia are frequently seen among adolescents than adult women [8, 9]. A study has also shown that increased trends of susceptibility among adolescents is associated with economic and social factors [10].

Adolescents have food preferences and poor eating behaviours, and for them, to make changes in their eating habits, they must believe that changes are possible within the context of their lifestyle and environment [11, 12]. Furthermore, adolescents who are pregnant may not have a stable food supply, food preparation skills, and facilities [13]. Therefore, appreciating issues that limit healthy eating along with challenges for nutrition education among pregnant adolescents are key when scheming and executing nutrition education interventions [14].

Nutritional education is aimed at transferring ample knowledge of healthy diet even though adequate knowledge may not be necessarily linked to practical dietary behaviour [15]. A study in Nigeria reported that 80.2% of adolescent girls had a poor level of nutritional knowledge towards reducing malnutrition [16]. Contrary to the study in Côte d'lvoire, the majority of the adolescents had good nutritional knowledge on nutritional health, a balanced diet, and hygiene [17]. In addition, studies have revealed that the dietary intake of adolescents was mostly plant-based food sources and high-energy snacks and beverages, with limited intake of fruits and vegetables and skipping of breakfast [18, 19]. Nutritional knowledge and dietary intake of this group could influence their nutritional status. For instance, studies carried out in Nigeria among female adolescents indicated that 23.4% and 9.4% of them were underweight and overweight/obese in 2014, respectively [20]; however, this trend changed to 38.2% underweight and 5.5% overweight/obese [21]. Additionally, a study in Ghana revealed that 7.1% and 54.5% of female adolescents were underweight and overweight/obese, respectively [22]. These studies identified nutritional deficiencies and knowledge gap among adolescents, however, not among pregnant adolescents. Again, pregnant adolescents are normally not included in national surveys, and their nutritional status around the world is limited [23]. Hence, this study investigated the nutritional knowledge and eating habits of pregnant adolescents in the Ledzokuku-Krowor Municipal in the Greater Accra Region of Ghana.

## 2. Materials and Methods

### 2.1. Study Site

Ledzokuku-Krowor municipality is one of the ten Municipalities in the Greater Accra Region of Ghana. It has an estimated population of 363,753. Ga-Adangbe is the main ethnic group in the area, with Christianity being the main religion of the people. There are three public hospitals providing antenatal services in the area. More than half (71.0%) of the people in the municipality are economically active, while 3.3% of the households in the area are engaged in agricultural production [24].

### 2.2. Study Population

The study involved pregnant adolescents (10–19 years) receiving antenatal care in the municipality and who have lived in the area for three or more months. However, data was collected from only those who agreed to be part of the study, and they and their guardians signed informed consent and assent forms.

### 2.3. Study Design

The study was cross-sectional and we used face-to-face interview technique to collect quantitative data on nutritional knowledge and eating habits from pregnant adolescents in the municipality between October and November 2019.

### 2.4. Sample Size

Four hundred and twenty-three participants involved in the study were determined using the formula established by Cochran and colleague for population-based cross-sectional studies [25]:

(1) $$\begin{array}{c} n=\;\frac{z^{2}\times p\left(q\right)}{d^{2}}, \end{array}$$

where *n* is the sample size to be determined, *z* is the *z*-score of 1.96 at 95% confidence level, *p* is the estimated proportion of an attribute that was present in the population (50% as the exact rate of good eating habits among pregnant adolescents is not known), *d* is the desired level of accuracy 5%, and *q* is 1 − *p*. Considering a 10% nonresponse rate among adolescents, the sample size was 423, to ensure the prevalence of the outcome variable falls within ±5% of the actual population coverage.

### 2.5. Sampling Method

Multistage sampling technique was applied to select the study participants. First, a total population purposive sampling was employed to select antenatal clinics in the three hospitals in the metropolis because they are government-owned facilities where pregnant women receive free antenatal care; hence, it is likely that more adolescent pregnant women will go there for services. Second, the total number of pregnant adolescents who have registered for antenatal care was taken and proportionately allocated the sample size to the facilities. To select the adolescents from the selected antenatal care clinics, the list of adolescents was sorted out from the antenatal care register, and we attached unique numbers to the names of the adolescents and wrote the numbers on a piece of paper, folded the papers, and placed them in a box. A neutral person was invited to pick a folded paper from the box, and the number written on the paper was traced to the attached name and repeated the process until we got the adolescents required.

### 2.6. Data Collection Tools and Procedure

Adolescent food habits checklist adapted from Johnson et al. [26] and a 24-hour dietary recall and food frequency questionnaire of the Food and Agricultural Organization of the United Nations [27] were used to collect food intake data, using a face-to-face interview procedure. We also used a set of questions to assess nutritional knowledge on dietary recommendations, source of nutrients, choosing everyday foods, and diet-disease relationships. We used the adolescents' contact information at the antenatal care clinics to track them to their houses for data collection. Research assistance was trained on the tools before data collection.

### 2.7. Data Processing and Analysis

The STATA 12.1 version software was used for data analysis. Participants who had a healthy response to all questions were classified as having a good eating habit. We further analysed and presented some details about eating habits of the adolescents as to whether they were eaten breakfast, snack after breakfast, lunch, snack after lunch, supper, snack after supper, eating at the right time, and frequency of the intake of various food groups and products. Again, twenty questions were used to evaluate adolescents' nutritional knowledge, where each correct response was rated 1 point and a wrong response was rated zero. Participants' overall nutritional knowledge was categorized using modified Bloom's cut-off point, as high if the score was between 80 and 100% (16–20 points), moderate if the score was between 50 and 79% (10–15 points), and poor if the score was less than 50% (<10 points). Therefore, sixteen or more points were classified as high knowledge, between ten and fifteen points were rated as moderate knowledge, and fewer than 10 points were regarded as low knowledge. Descriptive and inferential statistics comprising frequency, percentage, chi-square, and logistic regression were employed in analysing the data. All statistical analyses were considered significant at *p* value <0.05.

### 2.8. Ethical Issues

This study was reviewed and approved by the Ethical Review Committee of the Ghana Health Service with a protocol number GHS-ERC 113/10/16. Consent and assent were obtained from the adolescents and their guardians.

## 3. Results

### 3.1. Background Characteristics of Adolescents

Four hundred and twenty-three pregnant adolescents participated in the study. Their ages were between 12 and 19 years, with the mean age being 16.3 years (±1.5 sd). The majority (71.4%) of them were at the late adolescent stage (16–19 years). Four ethnic groups were involved with a comparative majority (44.0%) being Ga-Adangbes, and 76.1% of adolescents were Christians. About 16.5% of them did not go to school, while 53.7% and 29.8% had basic and secondary education, respectively. The majority (45.2%) of the adolescents were involved in petty trading as their main occupation. Almost all (91.0%) of them were in cohabiting relationships. Additionally, 75.9% of them were having their first pregnancy, and a comparative majority (41.6%) were receiving antenatal care from LEKMA Polyclinic (Table 1).

### 3.2. Nutritional Knowledge of Pregnant Adolescents

**Figure 1:**
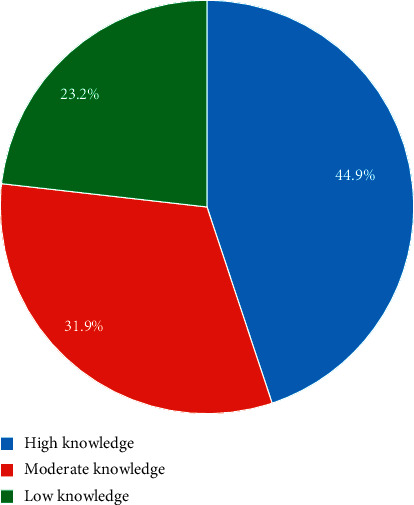
Nutritional knowledge of pregnant adolescents.

The results showed that 44.9% of the adolescents had high nutritional knowledge, while 31.9% and 23.2% had moderate and low nutritional knowledge, respectively (Figure 1).

### 3.3. Eating Habits of the Pregnant Adolescents

**Figure 2:**
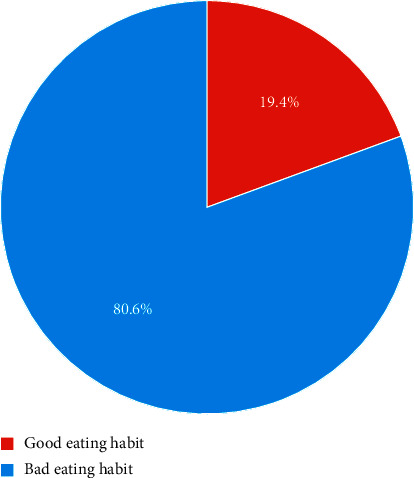
Eating habits of pregnant adolescents.

**Figure 3:**
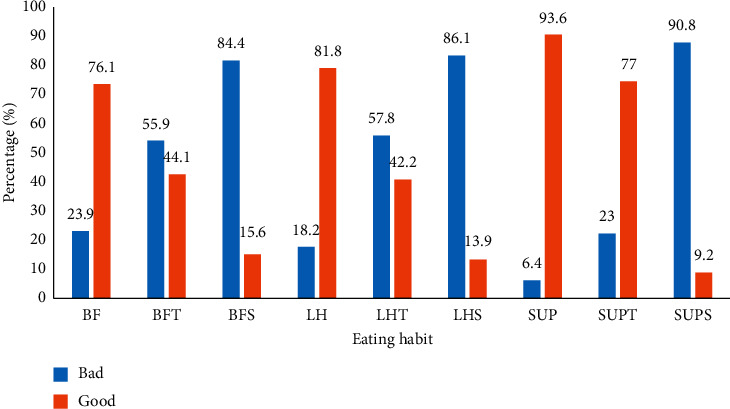
Eating habits of meals in a day. BF: breakfast; BFT: breakfast time; BFS: snack after breakfast; LH: lunch; LHT: lunchtime; LHS: snack after lunch; SUP: supper; SUPT: supper time; and SUPS: snack after supper.

The study showed that 19.3% of pregnant adolescents had good eating habits (Figure 2). The majority (76.1%, 81.8%, and 93.6%) of the adolescents do take breakfast, lunch, and supper/dinner daily; however, 55.9%, 57.8%, and 23.0% of them do not take their breakfast, lunch, and supper/dinner time, respectively. Furthermore, 84.4%, 86.1%, and 90.8% of the adolescents do not take snacks after breakfast, lunch, and supper/dinner, respectively (Figure 3).

### 3.4. Consumption of Food Groups and Products

The study revealed that 4.5% and 38.5% of adolescents have been consuming starchy roots/plantains and cereals/grains daily. In addition, 9.7%, 26.5%, and 42.1% of them do consume animal products, legumes/nuts/seeds, and fats/oil, respectively, every day. Furthermore, 3.8% of them have been consuming fruits and vegetables every single day. About 30%, 28%, and 23% of the adolescents are consuming carbonated drinks, pastries, and energy drinks, respectively, every day (Table 2).

### 3.5. Association between Eating Habits and Demographic Characteristics

The results reveal significant associations between good eating habits and the adolescent's ethnicity (*p*=0.017), educational level (*p*=0.014), occupation (*p*=0.016) , and whether the adolescent has had pregnancy before (*p*=0.021) using bivariate analysis. Furthermore, when multiple logistic regression analysis was used to test confounding effects, the analysis confirmed associations between ethnicity and eating habits and indicated that adolescents who were Ga-Adangbes (AOR: 1.71, 95% CI: 1.23–6.17, *p*=0.012) were more likely to be associated with good eating habits than those who were Akans, while adolescents who were Ewes (AOR: 0.63, 95% CI: 0.30–0.79, *p*=0.023) and Northerners (AOR: 0.18, 95% CI: 0.12–0.72, *p*=0.018) were less likely to be associated with good eating habits than those who were Akans. Additionally, adolescents who attained basic education (AOR: 0.31, 95% CI: 0.23–0.72, *p*=0.021) and secondary education (AOR: 0.55, 95% CI: 0.27–0.84, *p*=0.014) were less likely to be associated with good eating habits than those who did not go to school. Again, adolescents involved in petty trading as their main occupation (AOR: 3.33, 95% CI: 1.62–5.75, *p*=0.011) were more likely to be associated with good eating habits than those who were seamstress, while hairdressers (AOR: 0.39, 95% CI: 0.23–0.71, *p*=0.019) were less likely to be associated with good eating habits than those who were seamstress. Additionally, adolescents who were experiencing their first pregnancy (AOR: 4.56, 95% CI: 2.33–6.67, *p*=0.019) were more likely to be associated with good eating habits than those who were pregnant for the second or third time (Table 3).

## 4. Discussion

The majority (71.5%) of the pregnant adolescents were in their late adolescent (16–19 years) years, which corresponds with what was reported in the demographic and health survey [28]. Although 83.5% of the adolescents went to school, they could not go beyond the secondary level of education. This may be as a result of the early pregnancy, which forced them to terminate their education and can undesirably affect their knowledge of health and nutritional needs. A comparative majority (44.0%) of the adolescents were Ga-Adangbes; this is not surprising because the study took place in a Ga-Adangbe dominated region. Other ethnic groupings may have moved to the study area because most socioeconomic activities in the country occur there. The majority of Ghanaians are either Christians or Muslims [28]. Despite the strong religious inclination of the people, a high proportion (91.0%) of the adolescents were not legally married (cohabiting). Marriages of these kinds are not usually legally binding. Therefore, it affects the adolescent care capabilities, thereby limiting their ability to mobilise resource enough to ensure balance in diet.

This study showed that only 44.9% of pregnant adolescents had high knowledge of nutrition, which is less than the finding (61.1%) in South Africa [29]. However, the South Africa study was among in-school adolescents who were not pregnant. Again, only 48 adolescent girls were involved in the South Africa study, while this study involved 423 pregnant adolescents. This sample size and educational variations between the studies might explain the differences in nutritional knowledge. The present study also revealed that the majority of adolescents do take breakfast daily, which is contrary to the results of a similar study in Ghana [19]. Again, this study showed that most (93.6%) of adolescents do take supper every daily, which is higher than what was reported (73%) in Mexico [30]. Though, the level of civil status of pregnant adolescents in both countries may differ and might have contributed to the differences in daily supper intake, as indicated in the Mexican study. However, the present study did not assess this index variable. Nevertheless, both studies agree on the high consumption of three-square meals per day and lack of snack intake. The increased number of meals in a day could be attributed to the physiological changes that adolescents undergo during pregnancy [31], and the lack of snacks after meals may be due to financial constraints. Again, this study showed that the majority of pregnant adolescents do not take fruits and vegetables daily, which was similar to the findings of the studies conducted elsewhere [19, 32]. The inadequate intake of fruits and vegetables may be due to the lack of or low knowledge of their benefits or lack of financial means to buy fruits and vegetables.

The regenerative health and nutrition program under Ghana's Ministry of Health has recommended daily and increased consumption of fruits and vegetables to maintain good health and prevent degenerative diseases especially during pregnancy [33]; however, the frequency of fruits, vegetables, fish, meat, and egg intake recognised in this study was very low, although these foods contain nutrients that are of high biological value for pregnant women. This finding agrees with the study conducted among pregnant women in Malaysia [34]. The low consumption of these foods could be attributed to several reasons, including foods meant for worthy people, food taboos, beliefs, and myths, cost of foodstuffs, and financial constraints [35].

Educational level and age are risk factors for pregnant adolescents between 15 and 19 years old [36]. The present study also showed that educational level was significantly associated with good eating habits and is consistent with the results of a study carried out among in-school adolescents in Belgium [37]. However, the present study involves pregnant adolescents and indicated that those who have been to school before were less likely to have good eating habits than those who have never been to school, while the Belgium study involved in-school adolescents and showed that those with lower educational level have less healthy food habits than those with a higher level of education. Meanwhile, education within a specific environment such as schools and health facilities can be a valuable strategy to influence health and nutrition. School is an effective and efficient means to reach young people and provide their needs and interest [38–40]. However, nutrition education is not in the curricula of some grades and subject areas. Meanwhile, complete curricula incorporation can significantly increase students' knowledge of nutrition-related diseases, the nutrient value of foods, and fresh vegetable preference, with a decrease in soda drinks [41]. Yet, the present study showed that adolescents who did not go to school are more likely to be associated with good eating habits than those who attended school; hence, there is a need for further studies to ascertain contributory factors.

Furthermore, in the present study, it was revealed that occupation and ethnicity were major factors influencing pregnant adolescents' eating habits. This revelation agrees with a similar study in Nepal, which reported that unemployed women were two times more likely to be malnourished than employed women and women from Terai ethnic background were five times more likely to be malnourished than women in Mountain regions [42]. Studies in the Korea Republic and Israel revealed that high-class professionals and lower socioeconomic status had a high frequency of good eating habits and low diet quality, respectively [43, 44]. It could be that the more people earn, the more they have financial means to afford healthy diets. A study also revealed that ethnic differences in determinants for nutrition exist, which showed that adolescent culture is the dominant influence for this age group. Hence, modifications in constructs and types of foods are necessary for most adolescent nutrition education programs [45].

Although pregnant women receive nutrition education during antenatal visits, this study showed that those experiencing their first pregnancy were more likely to have good eating habits than those who have been pregnant before. Notwithstanding, a study reported that pregnant women were provided with little nutrition-related information in antenatal care and was perceived as presented in very general terms and focused on only food safety [46, 47]. Meanwhile, the midwife/clinician is a dependable source of nutrition-related information, yet limited nutrition knowledge and a lack of nutrition training impacted the capacity to provide adequate nutrition education [47]. Nevertheless, nutrition communication in antenatal care should be more focused on women's dietary habits and nutritional knowledge [46].

### 4.1. Study Limitation

The study could not assess the nutritional status of pregnant adolescents to compare with the nutritional knowledge and eating habits to determine the effect on the nutritional outcomes. Notwithstanding, we believe that the limitation cannot invalidate the findings of the study.

## 5. Conclusion

The eating habits among pregnant adolescents represent an important public health issue because of the long-term effects on the health of the mother and child. However, eating habits among adolescent pregnant women in the Ledzokuku-Krowor municipality were not encouraging, with only 19.4% of the adolescents having good eating habits despite many health facilities in the municipality rendering nutrition education to the people. The daily intake of fruits and vegetables and other nutrients by pregnant adolescents was not the best. All concerned stakeholders must come together and adopt different approaches to reduce adolescent pregnancies and to improve the eating habits of pregnant adolescents.

### 5.1. Recommendations

The municipal health authority should intensify public awareness and education on nutrition, especially adolescent nutrition.

The Ministry of Health and Ghana Health Service should give critical attention to adolescent nutrition as being given to adolescent reproductive health and other health-related interventions.

Efforts should be made by the government and other relevant stakeholders to include adolescent nutrition, to national surveys such as demographic and health studies, to measure indicators, and to generate nationally based nutrition data on adolescents.

A qualitative study should be conducted to identify the factors influencing eating habits among pregnant adolescents. Another survey should be carried out to assess the nutritional status of adolescents, using the ABCD assessment tool (Anthropometric, Biochemical, Clinical, and Dietary intake).

## Figures and Tables

**Table 1:** Demographic characteristics of adolescents.

Variable	Frequency	Percentage
Health facility		
Teshie Hospital	110	26.0
LEKMA Hospital	137	32.4
LEKMA Polyclinic	176	41.6

Age group (years)		
Early adolescent 12–15	121	28.6
Late adolescent 16–19	302	71.4

Ethnicity		
Akan	101	23.9
Ga-Adangbe	186	44.0
Ewe	54	12.8
Northerner	82	19.3

Religion		
Christian	322	76.1
Islam	89	21.1
Traditionalist	12	2.8

Educational level		
None	70	16.5
Basic	227	53.7
Secondary	126	29.8

Occupation		
Seamstress	121	28.6
Petty trader	191	45.2
Hairdresser	111	26.2

Marital status		
Legally married	38	9.0
Cohabitation	385	91.0

First pregnancy		
No	102	24.1
Yes	321	75.9

Number of children (*n* = 102)		
One	97	95.1
Two	5	4.9

**Table 2:** Consumption of various food groups and products.

Food type	1-2 days	3-4 days	5-6 days	Daily
Starchy roots/plantain	143 (33.8%)	67 (15.8%)	194 (45.9%)	19 (4.5%)
Cereals/grains	71 (16.8%)	92 (21.8%)	97 (22.9%)	163 (38.5%)
Animal products	91 (21.5%)	260 (61.5%)	31 (7.3%)	41 (9.7%)
Legumes/nuts/seeds	126 (29.8%)	72 (17.0%)	113 (26.7%)	112 (26.5%)
Fats/oil	67 (15.8%)	63 (14.9%)	115 (27.2%)	178 (42.1%)
Fruits	106 (25.1%)	281 (66.4%)	20 (4.7%)	16 (3.8%)
Vegetables	115 (27.2%)	275 (65.0%)	17 (4.0%)	16 (3.8%)
Soft drinks (carbonated drink)	156 (36.9%)	82 (19.4%)	58 (13.7%)	127 (30.0%)
Energy drinks	120 (28.4%)	116 (27.4%)	89 (21.0%)	98 (23.2%)
Pastries	84 (19.9%)	105 (24.8%)	116 (27.4%)	118 (27.9%)

**Table 3:** Association between respondents eating habits and demographic characteristics.

Variable	Bad eating habit *n* = 341 (%)	Good eating habit *n* = 82 (%)	COR (95% CI) *p* value^∗∗^		AOR (95% CI) *p* value
Health facility					
Teshie Community	87 (79.1)	23 (20.9)	—	0.088	—
LEKMA Hospital	110 (80.3)	27 (19.7)	0.92 (0.39–2.18)		—
LEKMA Polyclinic	144 (78.7)	32 (21.3)	1.81 (0.35–1.87)		—

Age group (years)					
12–15	98 (81.0)	23 (19.0)	—	0.198	—
16–19	243 (80.5)	59 (19.5)	1.64 (0.39–2.78)		—

Ethnicity					
Akan	78 (77.2)	23 (22.8)	—	**0.017**	—
Ga-Adangbe	143 (76.9)	43 (23.1)	1.65 (1.20–6.05)		1.71 (1.23–6.17) **0.012**
Ewe	43 (79.6)	11 (20.4)	0.62 (0.32–0.81)		0.63 (0.32–0.79) **0.023**
Northerner	77 (93.9)	5 (6.1)	0.54 (0.20–0.87)		0.18 (0.12–0.72) **0.018**

Religion					
Christian	251 (78.0)	71 (22.0)	—	0.096	—
Muslim	81 (91.0)	8 (9.0)	0.61 (0.55–3.25)		—
Traditionalist	9 (75.0)	3 (25.0)	2.02 (0.63–2.67)		—

Educational level					
No	37 (52.9)	33 (47.1)	—	**0.014**	—
Basic	202 (89.0)	25 (11.0)	0.29 (0.21–0.66)		0.31 (0.23–0.72) **0.021**
Secondary	102 (81.0)	24 (19.0)	0.47 (0.32–0.89)		0.55 (0.27–0.84) **0.014**

Occupation					
Seamstress	98 (81.0)	23 (19.0)	—	**0.016**	—
Petty trading	139 (72.8)	52 (27.2)	2.77 (1.21–5.95)		3.33 (1.62–5.75) **0.011**
Hairdresser	104 (93.7)	7 (6.3)	0.26 (0.14–0.63)		0.39 (0.23–0.71) **0.019**

Marital status					
Legally married	30 (78.9)	8 (21.1)	—	0.063	—
Cohabitation	311 (80.2)	74 (19.2)	0.31 (0.21–2.32)		—

First pregnancy?					
No	93 (91.2)	9 (8.8)	—	**0.021**	—
Yes	248 (77.3)	73 (22.7)	4.21 (2.21–6.96)		4.56 (2.33–6.67) **0.019**

Nutritional knowledge					
High	144 (75.8)	46 (24.2)	—	0.078	—
Moderate	110 (81.5)	25 (18.5)	0.54 (0.06–1.71)		—
Low	87 (88.8)	11 (11.2)	0.49 (0.03–1.98)		—

*p* value^∗∗^ is *p* value for the whole model.

## Data Availability

The data used to support the findings of this study can be made available from the corresponding author upon request.

## Abbreviations

LEKMA: Ledzokuku-Krowor municipality
GHS-ERC: Ghana Health Service-Ethical Review Committee
JHS: Junior high school
SHS: Senior high school
COR: Crude odds ratio
AOR: Adjusted odds ratio
BF: Breakfast
BFT: Breakfast time
BFS: Snack after breakfast
LH: Lunch
LHT: Lunchtime
LHS: Snack after lunch
SUP: Supper
SUPT: Supper time
SUPS: Snack after super.

## References

[1] Sawyer S. M., Afifi R. A., Bearinger L. H. (2012). Adolescence: a foundation for future health. *The Lancet*.

[2] Wagner C. L., Taylor S. N., Dawodu A., Johnson D. D., Hollis B. W. (2012). Vitamin D and its role during pregnancy in attaining optimal health of mother and fetus. *Nutrients*.

[3] Wu G., Imhoff-Kunsch B., Girard A. W. (2012). Biological mechanisms for nutritional regulation of maternal health and fetal development. *Paediatric and Perinatal Epidemiology*.

[4] Ibrahim S. A., Al-Halim O. A. F. A., Samy M. A., Mohamadin A. M. (2013). Maternal nutritional status and the risk of birth defects among Saudi women. *Nutrafoods*.

[5] King F. S., Burgess A., Quinn V. J., Osei A. K. (2015). *Nutrition for Developing Countries*.

[6] Castro-Quezada I., Román-Viñas B., Serra-Majem L. (2014). The Mediterranean diet and nutritional adequacy: a review. *Nutrients*.

[7] Hovdenak N., Haram K. (2012). Influence of mineral and vitamin supplements on pregnancy outcome. *European Journal of Obstetrics & Gynecology and Reproductive Biology*.

[8] Belete Y., Negga B., Firehiwot M. (2016). Under nutrition and associated factors among adolescent pregnant women in Shashemenne District, West Arsi Zone, Ethiopia: a community-based. *Journal of Nutrition & Food Sciences*.

[9] Chantrapanichkul P., Chawanpaiboon S. (2013). Adverse pregnancy outcomes in cases involving extremely young maternal age. *International Journal of Gynecology & Obstetrics*.

[10] Kearney M. S., Levine P. B. (2012). Why is the teen birth rate in the United States so high and why does it matter?. *Journal of Economic Perspectives*.

[11] Orloff N. C., Hormes J. M. (2014). Pickles and ice cream! food cravings in pregnancy: hypotheses, preliminary evidence, and directions for future research. *Frontiers in Psychology*.

[12] Feeley A. B. B. (2012). The impact of dietary habits and practices during adolescence on the risk of obesity: the birth to twenty cohort.

[13] Mahan L. K., Raymond J. L. (2016). *Krause’s Food & the Nutrition Care Process*.

[14] Wise N. J. (2015). Pregnant adolescents, beliefs about healthy eating, factors that influence food choices, and nutrition education preferences. *Journal of Midwifery & Women’s Health*.

[15] Gracey D., Stanley N., Burke V., Corti B., Beilin L. J. (1996). Nutritional knowledge, beliefs and behaviours in teenage school students. *Health Education Research*.

[16] Charles Shapu R., Ismail S., Ahmad N., Ying L. P., Abubakar Njodi I. (2020). Knowledge, attitude, and practice of adolescent girls towards reducing malnutrition in Maiduguri metropolitan council, Borno State, Nigeria: cross-sectional study. *Nutrients*.

[17] Jesson J., Kouakou E. K., Hardy-Johnson P. (2020). Adolescent nutrition and physical activity in low-income suburbs of Abidjan, Côte d’lvoire: the gap between knowledge, aspirations and possibilities. *Public Health Nutrition*.

[18] Ochola S., Masibo P. K. (2014). Dietary intake of schoolchildren and adolescents in developing countries. *Annals of Nutrition and Metabolism*.

[19] Doku D., Koivusilta L., Raisamo S., Rimpelä A. (2013). Socio-economic differences in adolescents’ breakfast eating, fruit and vegetable consumption and physical activity in Ghana. *Public Health Nutrition*.

[20] Omobuwa O., Alebiosu C., Olajide F., Adebimpe W. (2014). Assessment of nutritional status of in-school adolescents in Ibadan, Nigeria. *South African Family Practice*.

[21] Kola-Raji B. A., Balogun M. R., Odugbemi T. O. (2017). A comparative study of nutritional status of adolescents from selected private and public boarding secondary schools in Ibadan, South Western Nigeria. *Journal of Medicine in the Tropics*.

[22] Emmanuel K., Sackey A. S., Awere E. (2013). Assessment of the nutritional status of junior high school students-evidence from Mfantseman municipality of Ghana. *Science Journal of Public Health*.

[23] Cashman K. D. (2015). Vitamin D: dietary requirements and food fortification as a means of helping achieve adequate vitamin D status. *The Journal of Steroid Biochemistry and Molecular Biology*.

[24] Ghana Statistical Service (2014). 2010 population and housing census report.

[25] Snedecor G. W., Cochran W. G. (1980). *Statistical Methods*.

[26] FAO (2018). *Dietary Assessment: A Resource Guide to Method Selection and Application in Low Resource Settings*.

[27] Johnson F., Wardle J., Griffith J. (2002). The adolescent food habits checklist: reliability and validity of a measure of healthy eating behaviour in adolescents. *European Journal of Clinical Nutrition*.

[28] Ghana Demographic and Health Survey (2015). *Millennium Development Goals Indicators*.

[29] Oldewage-Theron W., Egal A., Moroka T. (2015). Nutrition knowledge and dietary intake of adolescents in Cofimvaba, Eastern Cape, South Africa. *Ecology of Food and Nutrition*.

[30] Guzmán-Mercado E., Vásquez-Garibay E. M., Troyo-Sanroman R., Romero-Velarde E. (2016). Food habits in Mexican pregnant adolescents according to their civil status. *Nutrición Hospitalaria*.

[31] Black R. E. (2001). Micronutrients in pregnancy. *British Journal of Nutrition*.

[32] da Mota Santana J., de Oliveira Queiroz V. A., Brito S. M., dos Santos D. B., Assis A. M. O. (2015). Food consumption patterns during pregnancy: a longitudinal study in a region of the North East of Brazil. *Nutricion Hospitalaria*.

[33] Amo-Adjei J., Kumi-Kyereme A. (2015). Fruit and vegetable consumption by ecological zone and socioeconomic status in Ghana. *Journal of Biosocial Science*.

[34] Mirsanjari M., Muda W., Manan W. (2016). Relationship between nutritional knowledge and healthy attitude and practice during pregnancy. *Borneo Science*.

[35] Gadegbeku C., Wayo R., Ackah-Badu G., Nukpe E., Okai A. (2013). Food taboos among residents at Ashongman-Accra, Ghana. *Food Science and Quality Management*.

[36] Smid M., Martins S., Whitaker A. K., Gilliam M. (2014). Correlates of pregnancy before age 15 compared with pregnancy between the ages of 15 and 19 in the United States. *Obstetrics & Gynecology*.

[37] Vereecken C., Maes L., Debacquer D. (2004). The influence of parental occupation and the pupils’ educational level on lifestyle behaviors among adolescents in Belgium. *Journal of Adolescent Health*.

[38] Aldinger C. E., Jones J. T. (1998). *Healthy Nutrition: An Essential Element of a Health-Promoting School*.

[39] Dixey R., Heindl I., Loureiro I., Pérez-Rodrigo C., Snel J., Warnking P. (1999). *Healthy Eating for Young People in Europe. A School-Based Nutrition Education Guide*.

[40] Perez-Rodrigo C., Aranceta J. (2003). Nutrition education in schools: experiences and challenges. *European Journal of Clinical Nutrition*.

[41] Amahmid O., El Guamri Y., Rakibi Y. (2020). Nutrition education in school curriculum: impact on adolescents’ attitudes and dietary behaviours. *International Journal of Health Promotion and Education*.

[42] Bhandari S., Sayami J. T., Thapa P., Sayami M., Kandel B. P., Banjara M. R. (2016). Dietary intake patterns and nutritional status of women of reproductive age in Nepal: findings from a health survey. *Archives of Public Health*.

[43] Shin Y. J., Park G. S. (1995). A study on eating habits of businessmen in urban areas. *Journal of the Korean Society of Food Culture*.

[44] Shahar D., Shai I., Vardi H., Shahar A., Fraser D. (2005). Diet and eating habits in high and low socioeconomic groups. *Nutrition*.

[45] Hoelscher D. M., Evans A., Parcel G., Kelder S. (2002). Designing effective nutrition interventions for adolescents. *Journal of the American Dietetic Association*.

[46] Garnweidner L. M., Pettersen K. S., Mosdøl A. (2013). Experiences with nutrition-related information during antenatal care of pregnant women of different ethnic backgrounds residing in the area of Oslo, Norway. *Midwifery*.

[47] Lee A., Newton M., Radcliffe J., Belski R. (2018). Pregnancy nutrition knowledge and experiences of pregnant women and antenatal care clinicians: a mixed methods approach. *Women and Birth*.

